# Synthesis of Water-Based Dispersions of Polymer/TiO_2_ Hybrid Nanospheres

**DOI:** 10.3390/nano5031454

**Published:** 2015-08-28

**Authors:** Lu Jin, Hua Wu, Massimo Morbidelli

**Affiliations:** Department of Chemistry and Applied Biosciences, Institute for Chemical and Bioengineering, ETH Zurich, 8093 Zurich, Switzerland; E-Mail: lu.jin@chem.ethz.ch

**Keywords:** hybrid, TiO_2_, nanocomposite, mini-emulsion, microchannel, intense turbulent shear

## Abstract

We develop a strategy for preparing water-based dispersions of polymer/TiO_2_ nanospheres that can be used to form composite materials applicable in various fields. The formed hybrid nanospheres are monodisperse and possess a hierarchical structure. It starts with the primary TiO_2_ nanoparticles of about 5 nm, which first assemble to nanoclusters of about 30 nm and then are integrated into monomer droplets. After emulsion polymerization, one obtains the water-based dispersions of polymer/TiO_2_ nanospheres. To achieve universal size, it is necessary to have treatments with intense turbulent shear generated in a microchannel device at different stages. In addition, a procedure combining synergistic actions of steric and anionic surfactants has been designed to warrant the colloidal stability of the process. Since the formed polymer/TiO_2_ nanospheres are stable aqueous dispersions, they can be easily mixed with TiO_2_-free polymeric nanoparticle dispersions to form new dispersions, where TiO_2_-containing nanospheres are homogeneously distributed in the dispersions at the nanoscale, thus leading to various applications. As an example, the proposed strategy has been applied to generate polystyrene/TiO_2_ nanospheres of about 100 nm in diameter.

## 1. Introduction

During the past decades, intensive studies have been focused on synthesis of hybrid nanomaterials that are composed of both inorganic and organic components [1,2,3,4,5]. Compared to the corresponding pure inorganic or organic materials, the hybrid composites have been demonstrated to have more widespread potential applications due to the improved mechanical [6], thermal [7], electrical [8,9], and optical properties [10,11,12]. Various morphologies of the hybrid nanomaterials have been developed, such as nano-rods, films, and particles [13,14,15,16,17,18].

Polymer/TiO_2_-based hybrid nanomaterials are one of the most common nanocomposites, which are widely applied in medicine [19,20,21,22], lithium batteries [23,24,25,26], UV-screening [27,28,29], sensors [30,31], and hybrid solar cell materials [32,33,34,35]. For most of these applications, the TiO_2_ nanoparticles (NPs) are typically used as fillers, which have to be homogeneously dispersed in the polymer matrix. The commonly-used technique for dispersing the TiO_2_ NPs in a polymer matrix is through direct blending of the TiO_2_ NP dispersion with the polymer solution. The major difficulty of this technique is to avoid agglomeration of the TiO_2_ NPs. To this aim, it has been proposed to use living free radical polymerization and to initiate the RAFT or ATRP polymerization from the surface of the TiO_2_ NPs in order to enhance the filler-polymer compatibility [36,37,38]. However, these approaches, though very efficient at the lab scale, are not attractive for large-scale industrial applications. Non-environmentally friendly organic solvents, typically used for both the TiO_2_ NP dispersion and the polymer solution, have to be evaporated in the final preparation of the hybrid materials and devices. In addition, the starting TiO_2_ NPs usually have irregular shape and broad size distribution, due to their unavoidable certain degree of agglomeration, which will affect the performance of the final hybrid materials.

**Scheme 1:**
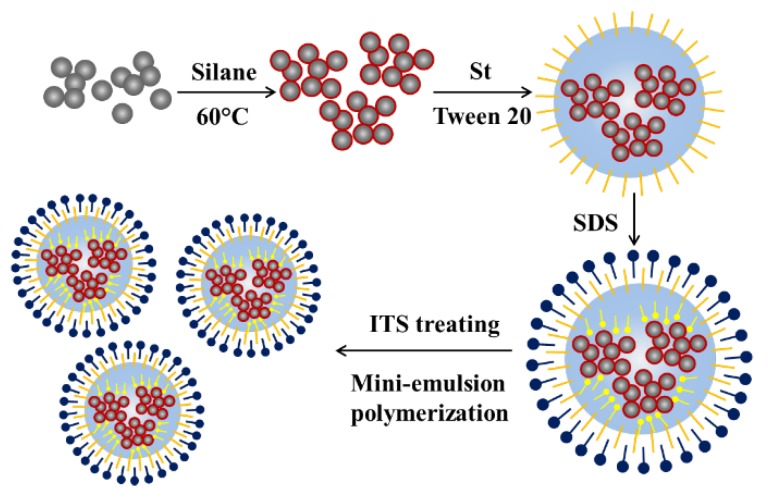
Schematic diagram showing the preparation procedure of TiO_2_/PS nanosphere.

In this work, we propose a different strategy to prepare polymer/TiO_2_-based hybrid nanomaterials, based on conventional emulsion polymerization, as shown in Scheme 1. In particular, we start with the synthesis of primary TiO_2_ NPs of ~5 nm. We first control their aggregation to form TiO_2_ nanoclusters (NCs) of ~30 nm, under intense turbulent shear (ITS) generated in a microchannel (MC) device. Then, we distribute the TiO_2_ NCs in the monomer droplets of a mini-emulsion, produced again using the ITS. These upon polymerization lead to monodisperse polymer/TiO_2_ nanospheres (NSs). The size and TiO_2_ content of the NSs can be varied by changing the monomer/TiO_2_ ratio, as well as the shear rate in the MC device. Since the produced polymer/TiO_2_ NSs are dispersed in water and stabilized by surface charges, they can be easily mixed with a TiO_2_-free polymeric NP dispersion to form a new dispersion, where TiO_2_-containing NSs are homogeneously distributed in the new polymer dispersion/matrix at the nanoscale. The concentration of the TiO_2_-containing NSs in the dispersion can be varied by changing the ratio of the two dispersions. Such obtained water-based dispersions can be applied to form hybrid materials and devices through either drying or coating or (injection and extrusion) molding (after drying and forming powders) or rotomolding. As an example, the proposed strategy has been successfully applied to prepare polystyrene/TiO_2_ NSs of about 100 nm in diameter.

## 2. Results and Discussion

### 2.1. TiO_2_ Primary Particles and Their Nanoclusters

**Figure 1:**
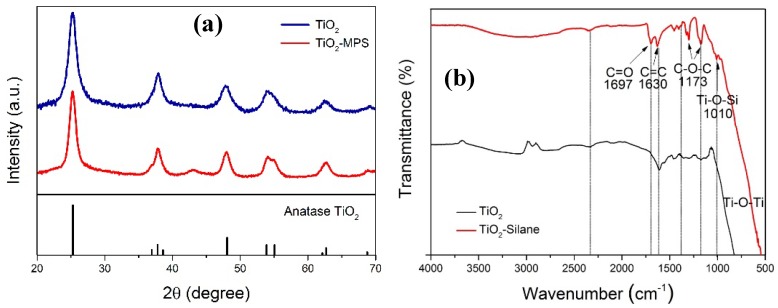
(**a**) XRD patterns of the synthesized TiO_2_ and MPS modified TiO_2_, where the standard differential peaks of anatase TiO_2_ (JCPDS No. 21-1272) is shown as vertical bars; (**b**) FT-IR spectra of the TiO_2_ NCs before and after surface modification with MPS.

**Figure 2:**
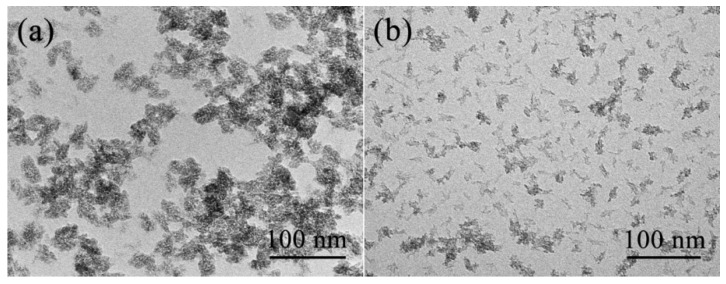
TEM images of the synthesized TiO_2_ nanoparticles in the form of nanoclusters (**a**) before and (**b**) after the ITS treatment.

Sol-gel methods involving thermal condensation reactions have been well-developed and widely applied to synthesize metal oxide NPs. Among them, the non-hydrolytic approach can better control the nucleation and growth of NPs at relatively low temperature, avoiding potential problems in the control of the nanoparticle morphology, surface composition, *etc.* [39,40]. In this work, we have applied a modified non-aqueous process to synthesize TiO_2_ primary NPs, as detailed in the Experimental section. Then, the obtained TiO_2_ primary NPs were precipitated using diethyl ether. After the mother liquor was decanted, the precipitated TiO_2_ primary NPs were thoroughly washed with pure ethanol to remove all residues and finally re-dispersed in ethanol with the help of sonication. The XRD analysis shown Figure 1a indicates that the obtained TiO_2_ is pure anatase. The state of the dispersion was investigated by both TEM imaging and DLS measurements. From the TEM picture shown in Figure 2a, the TiO_2_ primary NPs in the dispersion are agglomerated in the form of nano-clusters (NCs), with an average diameter, *D_p_* ≈ 200 nm. This is consistent with the average hydrodynamic diameter given by DLS and equal to 230 nm.

**Figure 3:**
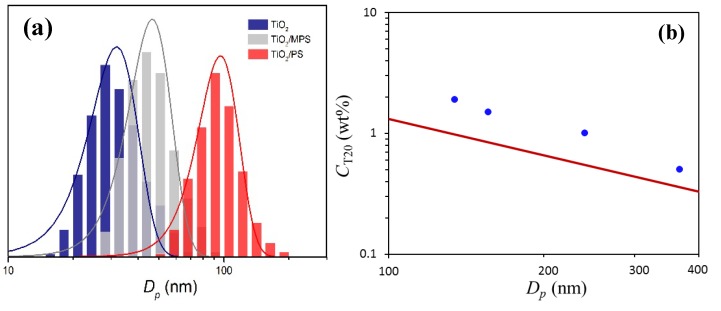
(**a**) Particle size distribution estimated from DLS in the cases of TiO_2_ NCs, MPS modified TiO_2_ and TiO_2_/PS NSs, respectively; (**b**) the experimental droplet diameter (*D_p_*) of the mini-emulsion at equilibrium at the TiO_2_/monomer mass ratio, 1:2, as a function of the overall concentration of Tween 20 (*C*_T20_). The continuous curve presents the amount of Tween 20 required to fully cover the surface of our mini-emulsion droplets, computed based on Equation (1).

Although TiO_2_ in the form of NCs is necessary in our work, the average diameter of 200 nm is too large, and also the morphology appears rather loose, as shown in Figure 2a. Thus, we need to reduce the size and tune the morphology of the TiO_2_ NCs. This can be done by intense sonication or by the ITS treatment [41]. In this work, we forced the TiO_2_ NCs dispersion to pass through the MC device three times at an inlet pressure of 340 bar, corresponding to an average shear rate of $\dot{\gamma }$ = 3.0 × 10^6^ s^−1^. After such ITS treatment, the hydrodynamic diameter of the TiO_2_ NCs in ethanol measured by the DLS becomes 30.2 nm, as shown in Figure 3a, substantially smaller than the initial value of 230 nm. The TEM images of the TiO_2_ NCs after the ITS treatment are shown in Figure 2b, which, when compared with Figure 2a, confirms the substantial reduction in the NCs size, and also a significant increase in compactness. It should be mentioned that the stable status of the TiO_2_ NCs in ethanol after the ITS treatment also results from surface positive charges. In particular, due to the acidic condition, the ethoxyl groups on the TiO_2_ surface are partially hydrolyzed, providing to the TiO_2_ NCs a positive ζ potential (+29.4 mV), thus improving the stability [42].

The surface modification of the TiO_2_ NCs in ethanol with 3-(trimethoxysilyl) propyl methacrylate (MPS) was carried out by adding excessive amount of MPS. Under the acidic conditions, as reported in the literature [43,44,45], the hydrolysis and condensation of organosilane are slow and controllable, and most of them are covalently bonded to the surface of nanoparticles. The TiO_2_ retains its anatase type after MPS modification, as shown in Figure 1a. In Figure 1b, the transmission FT-IR spectra of the TiO_2_ NCs with and without MPS modification are shown. It is seen that with MPS modification, peaks appear at 1697 cm^−1^, 1630 cm^−1^ and 1173 cm^−1^, corresponding to C=O, C=C, and C–O–C bonds, respectively, assigned to the MPS molecule. More importantly, a peak has been observed around 1010 cm^−1^, which specifically represents the Si–O–Ti bond, confirming the successful MPS modification of the TiO_2_ NC surface. It should be noted that the diameter of the TiO_2_ NCs after surface modification increases from the initial 30.2 nm to 46.9 nm, as shown in Figure 3a, based on DLS measurements. Two factors may contribute to this size increase: First, since each NC is constituted by several TiO_2_ primary particles, the MPS grafting occurs on the surface of all the TiO_2_ primary particles, thus separating them from each other and leading to certain “expansion” of the NC size. Second, since several grafting sites are present on each MPS molecule, they may lead to certain bridging among the NCs. However, such bridging should not occur significantly under acidic conditions [46].

The MPS-modified TiO_2_ NCs, after separation from the solvent and washing, were mixed with styrene and DVB monomers to form a TiO_2_ NC/monomer mixture, which will be dispersed in water to form mini-emulsions in the next step.

### 2.2. Control of the Stability of the TiO_2_ NC/Monomer Emulsion Droplets

Since our TiO_2_/PS hybrid nanospheres are produced through *in situ* polymerization, the stability of the TiO_2_ NC/monomer droplets in water becomes crucial for the success of the polymerization. To this aim, a two-step strategy was developed.

In the first step, we injected the TiO_2_ NC/monomer mixture into an aqueous Tween 20 solution at a given concentration, and followed by sonication with a probe sonicator. Due to the steric stabilization of Tween 20, as well as the positive surface charges from the hydrolysis of TiO_2_, the obtained mini-emulsion was very stable. However, after adding the initiator, KPS, to start the *in-situ* polymerization, aggregation of the TiO_2_ NC/monomer droplets occurred, leading to phase separation. This confirms that the stability of the mini-emulsion results from the synergistic role of both the steric stabilization of Tween 20 and the positive surface charges of TiO_2_. When the polymerization started after adding KPS, the resulting oligomer or polymer chains with anionic end groups (−OSO_3_^−^) would interfere with the positive surface charges of TiO_2_, leading to surface charge neutralization. Since the adsorbed Tween 20 molecules alone were unable to stabilize the emulsion droplets, phase separation occurred.

One may propose to increase the amount of steric stabilizer, Tween 20, so that it alone can stabilize the system. However, our experiments revealed that this is not a feasible solution. In particular, we have prepared mini-emulsions at four different concentrations of Tween 20. Figure 3b (filled circles) shows the measured diameter of the emulsion droplets (*D_p_*) at equilibrium as a function of the overall concentration of Tween 20, *C*_T20_. It is seen that *D_p_* decreases as *C*_T20_ increases. However, we observed that the above instability problem (phase separation) remains for all the four cases. In fact, we have computed the amount of Tween 20, *C*_T20,∞_, required to fully cover the surface of the emulsion droplets:

(1) $$C_{T20,\infty }=\frac{6\phi W_{T20}}{D_{p}A_{T20}N_{A}}$$

where ϕ is the volume fraction of the TiO_2_ NC/monomer mixture, *W*_T20_ (=1.23 × 10^3^ g·mol^−1^) is the molar mass of Tween 20, *A*_T20_ is the surface area occupied by one Tween 20 molecule, which is equal to 46.5 Å^2^, taken from the work of Nino and Patino [47], and *N_A_* is the Avogadro constant. The computed relation between *C*_T20,∞_ and *D_p_* for our system is shown in Figure 3b (continuous curve). It is seen that the *C*_T20,∞_ curve is under the experimental data, indicating that in all the four cases the added amount of Tween 20 is more than enough to fully cover the surface of the droplets. The excess amount of Tween 20 is the dissolved partition, equilibrating with the surface adsorption. It should be noted that the *C*_T20,∞_ curve depends on the *A*_T20_ value reported in the literature, which may be slightly different for the present system. Thus, the comparison in Figure 3b is only qualitative. However, the trend of the experimental data is almost in parallel to that of the *C*_T20,∞_ curve, which at least indicates that the density of Tween 20 on the surface is practically the same for the four cases. Thus, the stability of the given system cannot be improved by adding more Tween 20, which can only lead to reducing the droplet size.

An alternative of course is to use a cationic initiator for the polymerization [42] so that the charge neutralization does not occur during the polymerization. This is indeed a proper solution, but the obtained hybrid nanospheres would be positively charged. Considering that most of the polymeric nanoparticles produced industrially from emulsion polymerization are negatively charged, it would be difficult to homogeneously disperse our hybrid nanospheres with those polymeric nanoparticles due to electrostatic attraction, leading to destabilization. For example, such a homogeneous mixing of two different particle dispersions at the nanoscale is crucial for making nanocomposites through shear-driven hetero-aggregation [48]. Thus, we kept using KPS as the initiator but designed a second step to warrant the stabilization of the mini-emulsion droplets.

**Figure 4:**
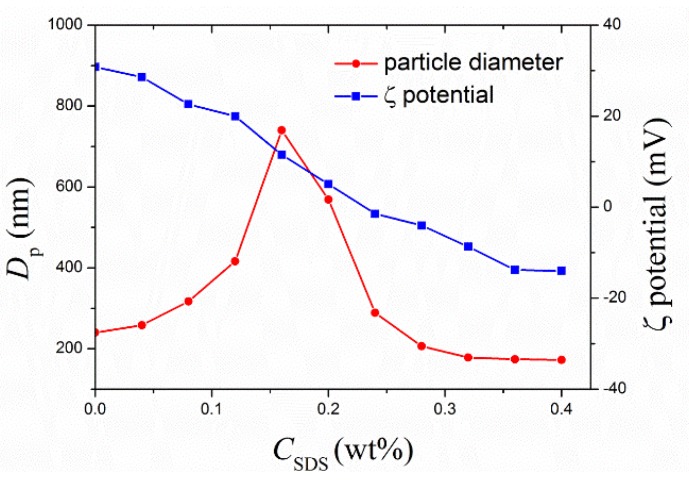
The hydrodynamic diameter and ζ-potential of mini-emulsion droplets, at the TiO_2_/monomer mass ratio, 1:2, as a function of the added amount of SDS, in the presence of Tween 20, *C*_T20_ = 1.0 wt%.

In the second step, the anionic surfactant, SDS, was introduced just after the Tween 20-only stabilized emulsion was formed, so as to have a synergistic stabilization played by both Tween 20 and SDS. In this case, it is evident that the added SDS also neutralizes the positive surface charges of TiO_2_. Then, in order to be sure that the added amount of SDS not only neutralizes the positive charges, leading to the charge inversion, but is also sufficient to stabilize the emulsion, we have monitored the hydrodynamic diameter and zeta-potential of emulsion droplets as a function of the added amount of SDS. The typical results are shown in Figure 4 at the concentration of Tween 20 added in the first step, *C*_T20_ = 1.0 wt%. It should be mentioned that during the experiments, when a given amount of SDS was added, the emulsion was treated by the probe sonicator at a power of 40 W for 10 min, and then the droplet diameter and ζ-potential were measured. We can see that there exist two stages:

(1) Initially, the TiO_2_ NCs/monomer droplets are positively charged, and with continuous addition of SDS, *C*_SDS_, the diameter of the droplets, *D_p_*, increases while the ζ potential decreases;
(2) When *D_p_* achieves a local maximum, which is around the location where the ζ potential approaches zero, it starts to decrease until reaching a plateau, and the corresponding ζ potential becomes negative and its absolute value increases with *C*_SDS_.

In Stage 1, as expected, the added SDS molecules affected the positive charges on the TiO_2_ surface, leading to charge neutralization, and the droplets are partially destabilized. It follows that the droplet diameter, *D_p_*, increases with *C*_SDS_, due to aggregation, while the ζ potential decreases. When the added SDS molecules fully neutralizes the positive charges, the ζ potential approaches zero, which leads to the lowest stability of the droplets, and the aggregation rate reaches the maximum, thus resulting in the largest droplet size. In Stage 2, further increase in the SDS concentration not only neutralizes the positive charges but also contributes to the stability of the droplets. Thus, the droplet diameter decreases with *C*_SDS_, and the ζ potential becomes negative and its absolute value increases. In Figure 4, when *C*_SDS_ > 0.3 wt%, the *D_p_* value decreases to reach a plateau. This may be related to the synergistic stabilization of Tween 20 and SDS, which involves complex competitive adsorption between Tween 20 and SDS on the surface. The plateau droplet diameter reached at the end is 170 nm, which is smaller than that (240 nm) before adding SDS, implying a lower density of Tween 20 on the surface required to stabilize the droplets due to the contribution of the adsorbed SDS.

It should be pointed out that the sequence of the above two steps is crucial for the successful stabilization of the emulsion system, *i.e.*, the droplets should be first stabilized by Tween 20 before the anionic SDS is introduced. Otherwise if SDS is used first, the TiO_2_ NCs/monomer droplets would immediately aggregate, leading to phase separation, and eventually their re-dispersion would become extremely difficult.

### 2.3. Improving the Distribution Uniformity of the Droplets by ITS

It is found that in the plateau region of *D_p_* in Figure 4, the polydispersity of the TiO_2_ NCs/monomer droplets is still rather high. For example, in the case of *C*_SDS_ = 0.36 wt%, from the DLS measurements, *D_p_* = 170 nm, and the polydispersity index, PDI = 0.22. To improve the uniformity of the droplet dispersion, we have applied the probe sonicator for another 10 min but at a power (280 W) substantially larger than that used for the experiments in Figure 4. The values of *D_p_* and PDI reduce to 133 nm and 0.18, respectively. In order to further improve the uniformity, we have applied the ITS technique which, as mentioned above, is more efficient to break up agglomerates, compared to sonication.

**Figure 5:**
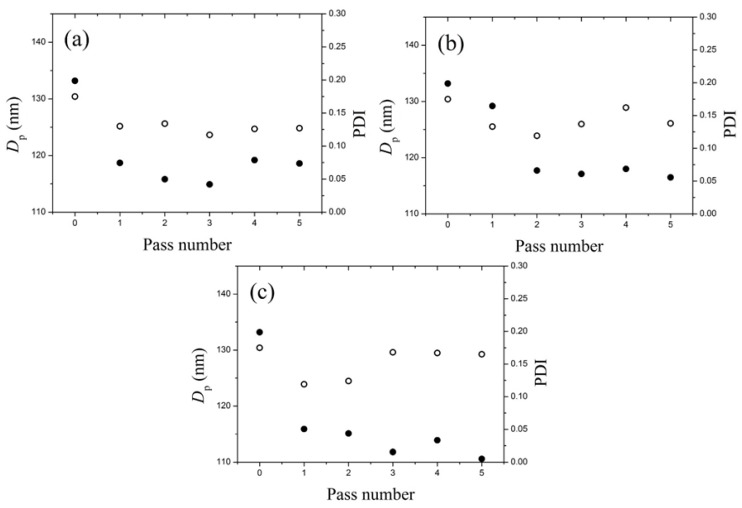
The average diameter (*D_p_*) and the polydispersity index (PDI) of the mini-emulsion droplets, at the TiO_2_/monomer mass ratio, 1:2, determined by DLS, as a function of the number of passes through the MC at three shear rates, (**a**) $\dot{\gamma }$ = 2.9 × 10^6^ s^−1^; (**b**) 3.4 × 10^6^ s^−1^ and (**c**) 3.9 × 10^6^ s^−1^ respectively.

Figure 5 shows the evolution of the *D_p_* and PDI values of the emulsion droplets with the number of passes through the MC at three shear rates, $\dot{\gamma }$ = 2.9 × 10^6^ s^−1^, 3.4 × 10^6^ s^−1^ and 3.9 × 10^6^ s^−1^, respectively. At the shear rate, $\dot{\gamma }$ = 2.9 × 10^6^ s^−1^, for the first three passes, both the *D_p_* and PDI values decrease with the pass number, and no significant change was observed with further passes. The reached minimum values of *D_p_* and PDI are ~115 nm and ~0.12, respectively. At the two larger shear rates, $\dot{\gamma }$ = 3.4 × 10^6^ s^−1^ and 3.9 × 10^6^ s^−1^ for the first three passes, the trends are very similar to that at $\dot{\gamma }$ = 2.9 × 10^6^ s^−1^, and the minimum values for *D_p_* and PDI are also equivalent. However, as the pass number increases, the PDI value tends to increase significantly, while the droplet diameter decreases. This becomes more evident in the case of Figure 5c of $\dot{\gamma }$ = 3.9 × 10^6^ s^−1^. A possible explanation of this behavior is, that at very high shear rates, the TiO_2_ NCs captured inside each droplet were restructured to become more compact [49], and some extra monomer at the periphery of the droplet were peeled off through shear erasion, forming very small (new) droplets without the TiO_2_ NCs. It follows that the polydispersity increases and the average droplet size decreases. Based on the above results, it can be concluded that in the explored range of the shear rate, $\dot{\gamma }$ ∈ [2.9 × 10^6^, 3.9 × 10^6^] s^−1^, in order to improve the distribution uniformity of the droplets, three to four passes through MC is adequate to guarantee sufficient breakage and homogeneous dispersion.

### 2.4. From Emulsion Droplets to Hybrid Nanospheres

Due to the combined effect of steric and electrostatic stabilization, the prepared mini-emulsions exhibit sufficient colloidal stability under mini-emulsion polymerization. However, it is worth pointing out that we noticed that, after several days, there was a vertical gradient concentration along the depth in latex. This phenomenon is inevitable since TiO_2_ has such a high density (4.23 g·cm^−3^) that the TiO_2_ NCs/monomer droplets tend to sediment, finally leading to phase separation. Therefore, the freshly prepared emulsion should undergo polymerization immediately after ITS treating.

**Figure 6:**
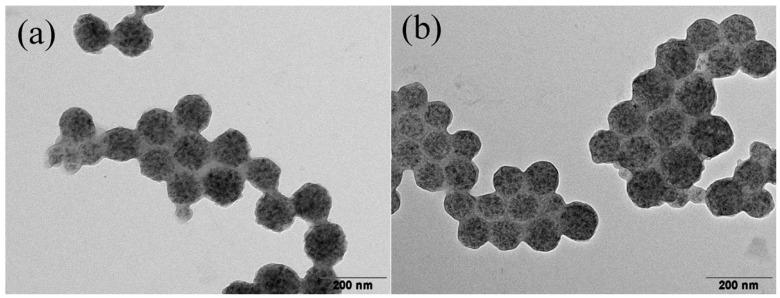
Typical TEM images of the hybrid TiO_2_/PS nanospheres, prepared at the TiO_2_/monomer mass ratio, (**a**) 1:1.5 and (**b**) 1:2.

Figure 6a,b show the TEM pictures of the hybrid nanospheres obtained after the polymerization, at the TiO_2_/monomer mass ratio equal to 1:1.5 and 1:2, respectively. It is seen that within each sphere, the TiO_2_ primary NPs are uniformly clustered and embedded in the cross-linked polymeric matrix, as indicated by the intensive dark spots in the TEM images. The average diameter of the nanospheres estimated from Figure 6a is *D_p_* ≈ 110 nm, consistent with the result obtained from the DLS measurement, 110 nm, as shown in Figure 3a. The sphericity of all these nanospheres is satisfactory, although the smoothness of the edges is not perfect. This may arise from the high mass and irregular shape of the TiO_2_ primary NPs within each sphere such that slight exposure of these rigid TiO_2_ primary NPs to the edge of the sphere would result in slightly irregular morphology. This is supported by the results in Figure 6b where, due to a smaller TiO_2_/monomer ratio (1:2), the edge of the nanospheres is smoother. In addition, we have also prepared the hybrid nanospheres at the TiO_2_/monomer ratio, 1:4, (pictures not shown), and found that not only the edge of the nanospheres becomes further smoother, but also their average diameter becomes smaller and the polydispersity is lower (*D_p_* = 107 nm and PDI = 0.02, from the DLS measurements). This is understandable since as the monomer mass increases in the emulsion droplets, the distance among the TiO_2_ NCs in the droplets increases, and it follows that the breakage of the droplets becomes easier. In fact, in the extreme case of pure monomer, the droplet diameter reduces to 41 nm under the same treating conditions. Therefore, by changing the TiO_2_/monomer ratio, one can tune the size and sphericity of the hybrid nanospheres. However, it should be noted that at the TiO_2_/monomer ratio smaller than 1:4, some nanospheres containing zero or one TiO_2_ NC appear.

## 3. Experimental Section

### 3.1. Materials

Titanium tetrachloride (TiCl_4_, 99.9%, trace metals basis), benzyl alcohol (>99.0%, GC), diethyl ether (>99.8%), silane coupling agent, MPS (>98%, nitrogen blushed), and potassium persulfate (KPS) were purchased from Sigma Aldrich, Switzerland, and used as received. Tween 20 and sodium dodecyl sulfate (SDS, >96.0%, GC) were provided by AppliChem and Fluka GmbH, Germany, respectively. Styrene monomer (St, >99.0%) (Sigma Aldrich) and the divinylbenzene (DVB) crosslinker (Merck) were purified by removing the inhibitor before polymerization. Ethanol of analytical grade obtained from Fluka and deionized water were used in all processes.

### 3.2. Synthesis of TiO_2_ Primary Nanoparticles and Surface Modification

A modified non-aqueous process was adopted to synthesize TiO_2_ primary NPs. This starts with the pre-alkoxylation of precursor (TiCl_4_) in ethanol, followed by its non-hydrolytic decomposition and TiO_2_ formation in benzyl alcohol [39,40]. In a typical preparation, 5 mL TiCl_4_ was dropwise added to a glass vial containing 25 mL ethanol at room temperature, which was constantly stirred for removing the generated gas. When a transparent yellow solution was formed, it was transferred to 100 mL of anhydrous benzyl alcohol to form a sol under continuous stirring. The reactor was kept at 80 °C in an oil bath equipped with a reflux condenser. After aging for more than 8 h, an opaque liquid was generated. The suspension was precipitated in sufficient amount of diethyl ether and centrifuged at 4500 rpm for 20 min with Multifuge 3S-R (Thermo Fisher, Switzerland), and the TiO_2_ precipitates were separated from the organic solvent by decantation. The obtained TiO_2_ product was thoroughly washed with pure ethanol by sonication-centrifugation treating, dispersed in 300 mL fresh ethanol and finally adjusted to acidic condition by adding droplets of 2 M HCl aqueous solution.

**Figure 7:**
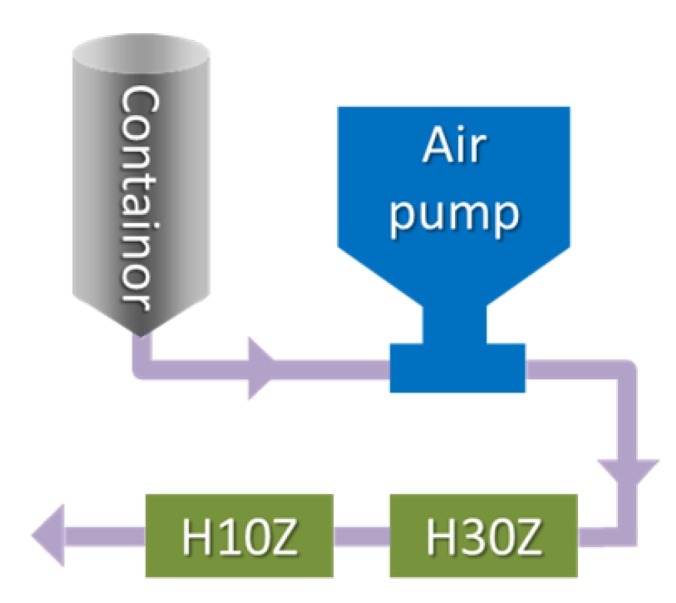
Sketch of the Microfluidizer setup with two interactive micro-channels, H30Z and H10Z, in series.

The as-prepared TiO_2_ dispersion was forced to pass through a MC device, Microfluidizer M-110Y (Microfluidics, USA), under intense turbulent shear (ITS) for three times. The setup of the MC device is shown in Figure 7, composed of two MCs in series, which are H30Z (with width, 200 μm) and H10Z (with width, 100 μm), operating at an inlet pressure between 300 bar and 600 bar. After the intense shear treatment, followed by adding 2 mL MPS, the TiO_2_ dispersion was heated to 60 °C under mild stirring, while kept refluxing, and maintained overnight. After centrifugation at 4500 rpm for 1 h, the supernatant containing excess MPS was discarded, and the remaining MPS-modified TiO_2_ precipitates were washed with fresh ethanol, before used for the next step.

### 3.3. Hybrid TiO_2_/PS Nanospheres through in-Situ Mini-Emulsion Polymerization

The still-wet MPS-modified TiO_2_ product was directly dispersed in the monomer, a mixture of styrene and DVB, where the mass ratio of styrene to DVB was 19:1, and the net mass ratio, TiO_2_/monomer (styrene + DVB), was set in the range from 1:1.5 to 1:4. Then, the dispersion was added to a Tween 20 aqueous solution and ultrasonically treated by a probe sonicator for 10 min to obtain a stable emulsion. Next, the ionic surfactant SDS was added to the system to invert the sign of the surface charges from positive to negative. To reduce the size of droplets to the desired value, we treated the emulsion again with the MC device under various conditions. The treated emulsion after adding the initiator, KPS, underwent *in situ* polymerization at 75 °C for more than 3 h to form the TiO_2_/PS nanospheres.

### 3.4. Characterization

Dynamic Light Scattering (DLS) and ζ-potential measurement: both the hydrodynamic diameter and ζ potential of the mini-emulsion before and after polymerization were measured by a Zetasizer Nano instrument (Malvern, UK). For a typical measurement, 10 µL dispersion was diluted by a 0.15 M SDS solution (to prevent any possible destabilization) to reach a particle volume concentration smaller than 1.0 × 10^−4^.

Fourier Transform Infrared Spectrum (FT-IR): the surface chemical structure of the modified TiO_2_ particles was characterized using an infrared spectrophotometer, VERTEX 70 (Bruker, Germany). The sample was extracted with refluxing acetone overnight, dried at 60 °C to remove the solvent, and the spectra recording was proceeded in the spectral range of 4000–400 cm^−1^ at a resolution of 4 cm^−1^.

Transmission Electron Microscopy (TEM): the TEM images of the particles were taken using a FEI Morgagni 268, operated at 120 kV, equipped with an Orius SC1000 CCD camera. For the characterization of the TiO_2_ nanoparticles, pure ethanol was used to redisperse the wet precipitate. A drop of the dispersion was deposited onto a copper grid covered by an amorphous carbon film. For the hybrid TiO_2_/PS nanospheres, the dispersion was diluted with deionized water and pipetted on the grid. The excess liquid was removed through a filter paper and the grids were allowed to dry prior to measurement.

## 4. Conclusions

We have developed a procedure for preparing TiO_2_/polystyrene (PS) hybrid nanospheres, where the TiO_2_ primary nanoparticles of ~5 nm are clustered and homogeneously distributed in the host PS matrix. As illustrated in Scheme 1, a procedure combining utilization of both steric and anionic surfactants, together with application of intense turbulent shear, has guaranteed the highly efficient and controllable preparation of the TiO_2_/monomer emulsion and, consequently, the synthesis of rather monodisperse hybrid nanospheres through *in situ* polymerization. The synthesized hybrid nanospheres have an average diameter of ~100 nm, with a hierarchical structure starting with the initial TiO_2_ primary nanoparticles (~5 nm), which first assemble to nanoclusters (~30 nm) and then are integrated into the nanospheres. Compared to the other methodologies reported in the literature, the present strategy leads to controllable size, narrow distribution, and large incorporated TiO_2_ fractions of the hybrid nanospheres. The non-harsh experimental conditions and ease of operation allow this strategy to be particularly advantageous in large-scale fabrication. The obtained TiO_2_/PS hybrid nanospheres can find various applications in organic semiconductor, self-cleaning coating and electronic devices. We expect that the developed strategy can be potentially applied to synthesize hybrid nanospheres of different metal oxides and polymers, with sizes in a wide range around the 100 nm investigated in this work.
